# Effect of Pre-Sulfidization on the Octadecyl Amine Adsorption on the Smithsonite Surface and Its Flotation

**DOI:** 10.3390/molecules29163921

**Published:** 2024-08-20

**Authors:** Mengtao Wang, Haobin Wei, Saizhen Jin, Guofan Zhang

**Affiliations:** 1 Faculty of Land Resource Engineering, Kunming University of Science and Technology, Kunming 650093, China; wang.mt@foxmail.com; 2 School of Minerals Processing and Bioengineering, Central South University, Changsha 410083, China; 3 Shandong Bureau of China Metallurgical Geology Bureau, Jinan 250014, China; whbhonor@163.com

**Keywords:** pre-sulfidization, flotation, smithsonite, adsorption mechanism, zinc oxide ore

## Abstract

The low-grade zinc oxide ore was sulfidized to increase the efficiency of flotation, but the effect of pre-sulfidization on the adsorption mechanism of octadecyl amine (ODA) on the smithsonite surface is currently unclear. In this study, the effect of pre-sulfidization on the adsorption mechanism of ODA and the flotation behavior was studied using smithsonite and pre-sulfidized smithsonite as the samples by zeta potential, contact angle measurement, total organic carbon analyzer (TOC), quartz microcrystalline balance (QCM), Fourier transform infrared spectroscopy (FTIR), X-ray photoelectron spectroscopy (XPS), and micro-flotation tests. Micro-flotation tests showed that the pretreatment of sulfidization could improve the floatability of smithsonite. Zeta potential and contact angle measurements demonstrated that pre-sulfidization could favor the adsorption of ODA, which is further confirmed by the adsorption tests of ODA using TOC and QCM. Furthermore, FTIR and XPS analysis showed that pre-sulfidization changes the adsorption mode of ODA, changing it from physical adsorption to chemical adsorption. These results suggested that the favorable effect of pre-sulfidization on the adsorption of ODA and the flotation of smithsonite might provide important guidance for industrial application.

## 1. Introduction

Zinc is an important basic metal material in economic construction, which is widely applied in metal protection, alloy manufacturing, zinc chemical products, energy reserves, and other fields [1,2,3]. Zinc oxide minerals are an abundant and important mineral source for extracting zinc, and the annual production of these ores could exceed 10% of the global production of zinc metal [4], and smithsonite (ZnCO_3_) is the most valuable in the industry [5,6]. However, the current mineral processing technology on low-grade zinc oxide ore is still unable to meet the demand for its efficient development and utilization, resulting in a large waste of zinc resources [7,8]. Therefore, it is of great importance for the supply security of zinc resources to develop an economic and efficient beneficiation scheme for low-grade zinc oxide ores. Due to the complex properties of low-grade zinc oxide ore, it is often difficult to deal with it directly by metallurgical methods and requires prior enrichment by flotation.

Amine surfactants can hydrolyze cationic groups (-NH_3_^+^) in water, which can adsorb with the mineral surface, and the hydroxyl part has strong hydrophobicity, making the mineral surface hydrophobic. Therefore, it is a class of cationic surfactants commonly used in mineral flotation. In the reverse flotation desilication of iron ore, amine collectors can selectively adsorb on the quartz surface to make the quartz hydrophobic, and the longer the alkyl carbon chain, the better the hydrophobicity [9]. Compared with traditional amine collectors, the oxidization of amines provides eco-friendly collectors and significantly improves the selective adsorption on the surface of quartz [10]. Due to the difference in electrostatic effect, tertiary amines show better floating recovery for kaolinite compared to diaspore, and the collecting powers are closely related to the substituent groups of amine collectors, exhibiting different inductive electronic and steric effects [11]. The sulfidization flotation, sulfidization with Na_2_S followed by treatment with amine as the collector, is one of the most common methods to recover zinc oxide minerals. However, the main challenge of the methods is lower selectivity and higher reagent consumption.

A great deal of research has been carried out on the action mechanism between amine collectors and zinc oxide minerals. Armac C and Armac T alkylamine acetates could be used as the collectors for smithsonite and successfully flotated smithsonite from the tailings of cerussite flotation circuit [12]. The pretreatment of sodium sulfide could enhance the adsorption ability of dodecyl amine on smithsonite, and the activation mechanism is that the adsorption of HS^−^ ions on the surface of smithsonite reduces its surface zeta potential, which is conducive to the electrostatic adsorption of dodecyl amine [13]. Chen et al. studied the sulfidization process of smithsonite by density functional-based tight binding (DFTB+) calculation. The results showed that the strong hydration of the (101) surface in smithsonite obstructed its interaction with amine collectors, and HS^−^ ions could form the structure of Zn-SH-SH with zinc active sites on the smithsonite (101) surface in the low-sodium sulfide dosage, hindering the interaction between the amine collector and the zinc sites, and the smithsonite (101) surface forms ZnS in the high-sodium sulfide dosage, which is conducive to the adsorption of amine collectors [14,15]. Deng et al. also used ToF-SIMS research results to show that amine collectors can hardly be adsorbed on the surface of smithsonite, but they are chemically adsorbed on the surface of smithsonite after sulfidization by forming N-S-Zn, N-Zn-S, or Zn-N-S [16]. Luo et al. found that the thickness of the sulfide film formed on the smithsonite surface played an important role in smithsonite’s floatability in the dodecyl amine system, and smithsonite began to float since its thickness reached 7.79 nm and exhibited a good floatability when its thickness increased to 12.13 nm [17]. Currently, few studies have been found to systematically study the effect of sulfidization on the adsorption of amine collectors, and most of the studies were in the presence of sodium sulfide, which might influence the adsorption of amine collectors. Avoiding the interference of sodium sulfide is very crucial to reveal in depth the effect of sulfidization on the adsorption of amine collectors.

In this study, the effect of sulfidization on the adsorption behavior of octadecyl amine on the smithsonite surface and its flotation was systematically investigated. Micro-flotation tests, zeta potential, and contact angle measurements were conducted to research the effect of sulfidization on the floatability and surface properties of smithsonite, respectively. TOC and QCM measurements were made to investigate the effect of sulfidization on the adsorption amounts of ODA. The further interaction mechanism of sulfidization on the adsorption mode of ODA was further studied by FTIR and XPS analysis.

## 2. Results and Discussion

### 2.1. Effect of Sulfidization on the Surface Properties of Smithsonite

To avoid the interference of Na_2_S, 5 × 10^−4^ mol/L Na_2_S was added and agitated for 5 min, after settling for 30 s; then, solid–liquid separation was used to obtain sulfidized smithsonite. The effect of sulfidization on the surface properties was characterized by SEM-EDS, FTIR, and XPS.

**Figure 1:**
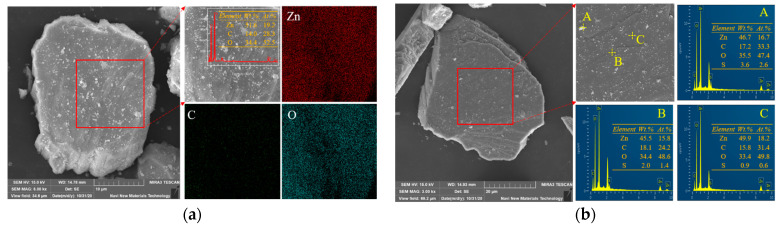
SEM-EDS of bare smithsonite (**a**) and sulfidized smithsonite (**b**).

SEM-EDS was used to observe the surface morphology and elemental changes of smithsonite and sulfidized smithsonite, and the apparent morphology and photoelectron spectral distribution of bare smithsonite and sulfidized smithsonite are shown in Figure 1a and Figure 1b, respectively.

As shown in Figure 1a, the surface of smithsonite is relatively rough with fracture zones. A large number of fine-grained smithsonite particles adhere to the mineral surface, which may be due to the low hardness of smithsonite itself, which can be easily crushed. The micro-fine-grained minerals have strong specific surface energy, which can be adsorbed on the surface of the coarse-grained smithsonite. The distribution of elements on the surface of smithsonite was further analyzed by EDS, and only the characteristic absorption peaks of Zn, C, and O appeared in the surface scanning spectra, and these three elements were uniformly distributed on the surface of smithsonite with the content of Zn, C, and O atoms of 19.2%, 28.3%, and 52.5%.

Figure 1b shows the SEM-EDS of the sulfidized smithsonite; the apparent morphology of smithsonite after sulfidization is relatively complete and has not changed significantly, indicating that the sulfidization treatment has not had a significant impact on the bulk structure of smithsonite. The surface of smithsonite after sulfidization is relatively neat, and the amounts of fine particles attached are significantly reduced, which may be related to the strong dispersing effect of sodium sulfide. The surface of smithsonite after sulfidization was further analyzed using the EDS energy spectrum, and it was found that the characteristic peak of the S element appeared in the photoelectron energy spectrum of smithsonite after sulfidization, indicating that sulfide was generated on the surface of smithsonite after sulfidization. The sulfur content of smithsonite at different sites after sulfidization is quite different. The fine particles on the surface of the sample have the largest sulfur content, and the atomic content of S reaches 2.6%; the atomic content of S at the edge of the crack is second, which is 1.4%; and the sulfur content at the smooth and dense area is the lowest, which is only 0.6%, indicating that the sulfidization products generated on the surface of smithsonite are unevenly distributed, and the fracture zone on the surface and the surface of fine particles may be more prone to sulfidization reaction.

**Figure 2:**
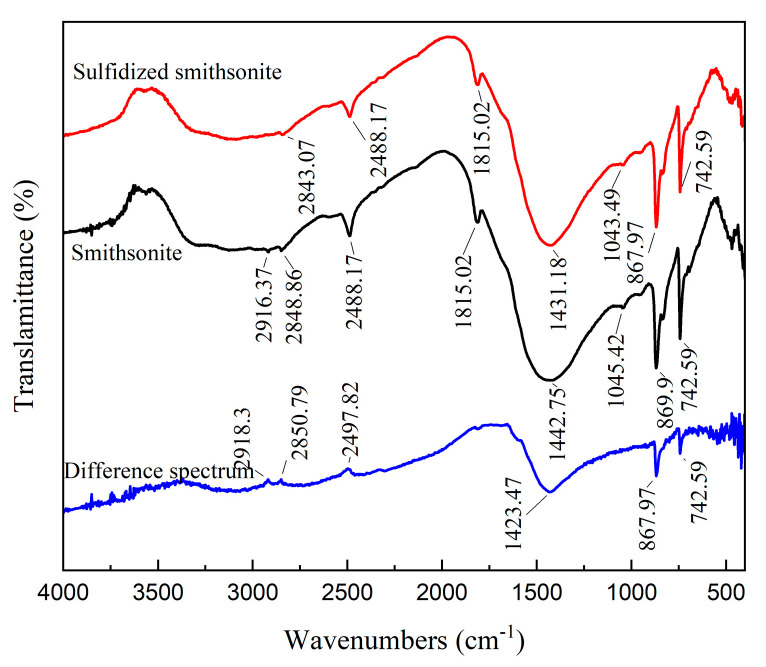
FTIR spectrum of bare smithsonite and sulfidized smithsonite.

Figure 2 shows the FTIR spectrum of smithsonite before and after sulfidization. The absorption peaks on the surface of smithsonite are mainly distributed at 2916.37 cm^−1^, 2848.86 cm^−1^, 2488.17 cm^−1^, 1815.02 cm^−1^, 1442.75 cm^−1^, 1045.42 cm^−1^, 869.9 cm^−1^ and 742.59 cm^−1^. According to relevant literature [18,19], the absorption peaks at 1442.75 cm^−1^, 869.9 cm^−1^ and 742.59 cm^−1^ are the characteristic peaks of CO_3_^2−^ ions in smithsonite, among which the absorption peak at 1442.75 cm^−1^ is the asymmetric stretching vibration absorption peak of CO_3_^2−^ ions, and the absorption peaks at 869.9 cm^−1^ and 742.59 cm^−1^ are the out-of-plane and in-plane bending vibration absorption peaks of CO_3_^2−^ ions, respectively. The absorption peak at 2488.17 cm^−1^ is the combined frequency peak of the CO_3_^2−^ ions asymmetric stretching vibration absorption peak and the out-of-plane bending vibration absorption peak, while the absorption characteristic peaks at 2916.37 cm^−1^, 2848.86 cm^−1^, and 1815.02 cm^−1^ are also considered to be harmonic peaks or combined frequency peaks. The absorption peak at 1045.42 cm^−1^ can be considered to be the vibration absorption peak of the zinc metal hydroxyl compound (Zn-OH) on the surface of smithsonite.

No new characteristic absorption peaks appear in the FTIR of smithsonite after sulfidization treatment, which may be because the generated sulfidization product is similar to the infrared spectrum of sphalerite, and the characteristic absorption peak appears below 400 cm^−1^. After sulfidization, the asymmetric stretching vibration absorption peak of CO_3_^2−^ ions in smithsonite decreased from 1442.75 to 1431.18 cm^−1^, and the harmonic peak or combined frequency peak at 2916.37 cm^−1^ disappeared, indicating that the sulfidization treatment had a certain effect on the surface structure of smithsonite. Further, taking the characteristic absorption peaks at 2488.17 cm^−1^ and 1815.02 cm^−1^ as reference peaks, the infrared spectrum of smithsonite before and after sulfidization was processed by the peak difference. It can be seen that after sulfidization, the relative peak intensity of the characteristic absorption peaks of CO_3_^2−^ ions at 1442.75 cm^−1^, 869.9 cm^−1^, and 742.59 cm^−1^ increased, while the relative peak intensity of the harmonic peak or combined frequency peak on the surface of smithsonite decreased, indicating that the sulfidization treatment changed the chemical environment of the zinc metal particles on the surface of smithsonite.

**Figure 3:**
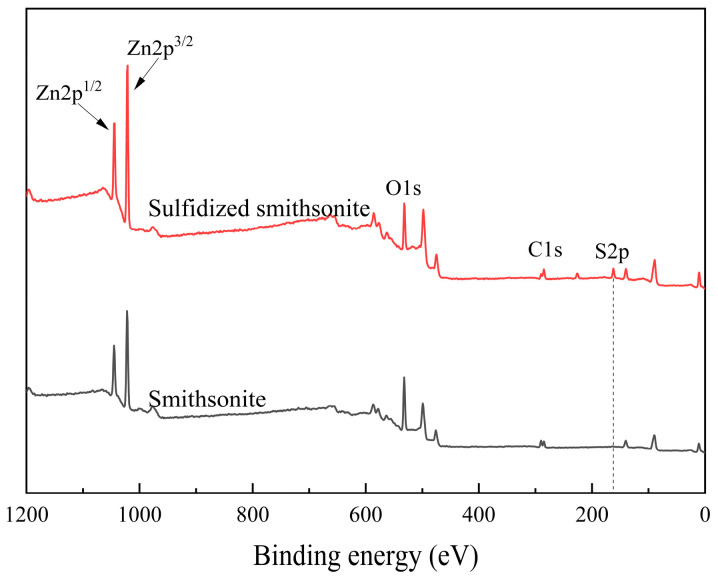
XPS spectra of bare smithsonite and sulfidized smithsonite.

The XPS spectrum of the smithsonite before and after sulfidization and the surface element atomic abundance and binding energy changes are shown in Figure 3 and Table 1, respectively. Combining the results in Figure 3 and Table 1, it can be seen that in the XPS spectrum of the surface of sulfidized smithsonite, a characteristic peak with a binding energy of 161.86 eV attributed to S2p appears, and the surface sulfur atom abundance reaches 14.29%. It shows that sulfide ions are adsorbed on the surface of smithsonite and form sulfide products.

Compared with bare smithsonite, the Zn atomic abundance on the surface of smithsonite increased significantly after sulfide treatment, from 20.96% to 29.89%, and the Zn2p binding energy decreased from 1022.04 to 1021.59 eV, a decrease of 0.63 eV, further indicating that after sulfidization treatment, the chemical environment of zinc sites on the surface of smithsonite has changed significantly. Compared with the changes in zinc atoms, sulfidization has less influence on the changes in C and O binding energies on the surface of smithsonite. Sulfidization has a greater impact on the relative abundance of atoms. The atomic abundances of C1s and O2s decreased by 5.17% and 18.05%, respectively. This may be related to the generation of sulfide products on the surface of smithsonite. The sulfide products cover the surface of smithsonite, and the relative abundance of oxygen atoms decreases.

**Figure 4:**
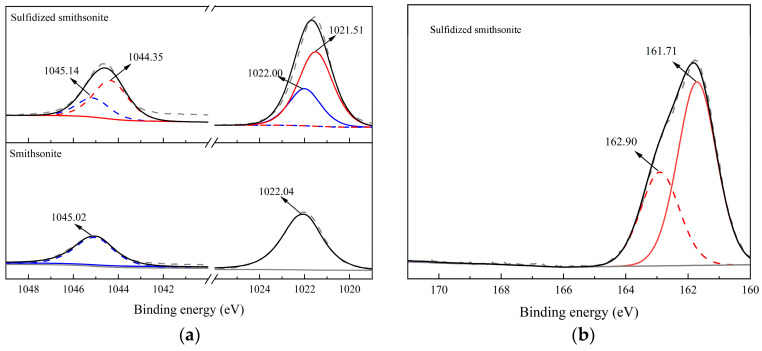
High-resolution spectra of Zn2p (**a**) and S2p (**b**) of bare smithsonite and sulfidized smithsonite.

In order to deeply analyze the properties of sulfide products and deeply study the impact of sulfide on the chemical state of elemental elements on the surface of smithsonite, high-resolution scans of Zn2p on the surface of smithsonite before and after sulfide and S2p after sulfide were carried out, and the fine spectra obtained were carried out. The test results after peak fitting processing are shown in Figure 4a and Figure 4b, respectively.

Figure 4a shows high-resolution spectra of Zn2p of smithsonite before and after sulfidization, from which it can be seen that the binding energies of the Zn2p^3/2^ and Zn2p^1/2^ peaks on the surface of smithsonite are located at 1022.04 eV and 1045.02 eV, respectively, which should be attributed to the characteristic peaks of ZnCO_3_. The Zn2p binding energy on the surface of smithsonite changed significantly after sulfidization, and the split-peak fitting process revealed that in addition to the characteristic peaks of Zn2p^3/2^ and Zn2p^1/2^ located at 1022.00 eV and 1045.14 eV attributed to smithsonite, a pair of Zn2p^3/2^ and Zn2p^1/2^ characteristic peaks located at 1021.51 eV and 1044.35 eV were newly appeared on the surface of the samples. As shown in the relevant literature, the peaks should be attributed to ZnS, indicating that the sulfide products generated on the surface of zincite after sulfidization are ZnS substances. The zinc atoms on the surface of smithsonite after sulfidization mainly exist in the form of ZnS and ZnCO_3_, and according to the calculation of the peak area of the two, the proportion of ZnS and ZnCO_3_ on the surface after sulfidization is 68.31% and 31.69%, respectively, which indicates that the sulfidization treatment can generate a large number of ZnS substances on the surface of zincite. Figure 4b is the high-resolution spectra of S2p of sulfidized smithsonite. The S2p spectrum on the surface of sulfidized smithsonite can be separated into a pair of sub-peaks. The binding energies of S2p^3/2^ and S2p^1/2^ sub-peaks are located at 161.71 eV and 162.90 eV, respectively. According to the relevant literature, this peak position belongs to the characteristic peak of ZnS. Therefore, it is further concluded that the sulfidization product on the surface of sulfided smithsonite is ZnS.

### 2.2. Effect of Sulfidization on the Flotation Behavior of Smithsonite

**Figure 5:**
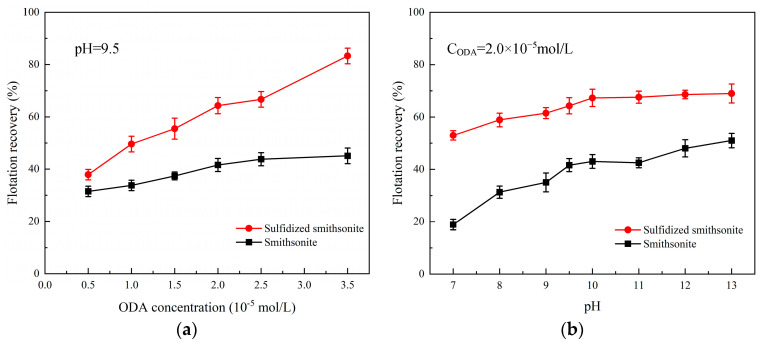
Effect of ODA concentration (**a**) and pulp pH (**b**) on the flotation recovery of bare smithsonite and sulfidized smithsonite.

In order to investigate the effect of sulfidization on the floatability of smithsonite in the presence of an octadecyl amine (ODA) collector, the flotation recovery of bare smithsonite and sulfidized smithsonite as a function of ODA concentration at pH 9.5 was tested, and the results are shown in Figure 5.

It could be seen from Figure 5 that by raising the concentration of ODA from 0.5 × 10^−5^ to 3.5 × 10^−5^ mol/L, the flotation recovery of bare smithsonite slightly increased from 30% to 45% in the presence of ODA alone, while the flotation recovery of smithsonite was only 41.6% at the ODA concentration of 2 × 10^−5^ mol/L, indicating that the flotation behavior of smithsonite using ODA as the collectors was poor. However, sulfidization has changed this situation, and the floatability of sulfidized smithsonite with its recovery from 38% to 82% was significantly improved after the pretreatment of sulfidization before ODA. The flotation recovery can be increased to 64.3% with pretreatment of sulfidization at the same ODA concentration of 2 × 10^−5^ mol/L.

**Figure 6:**
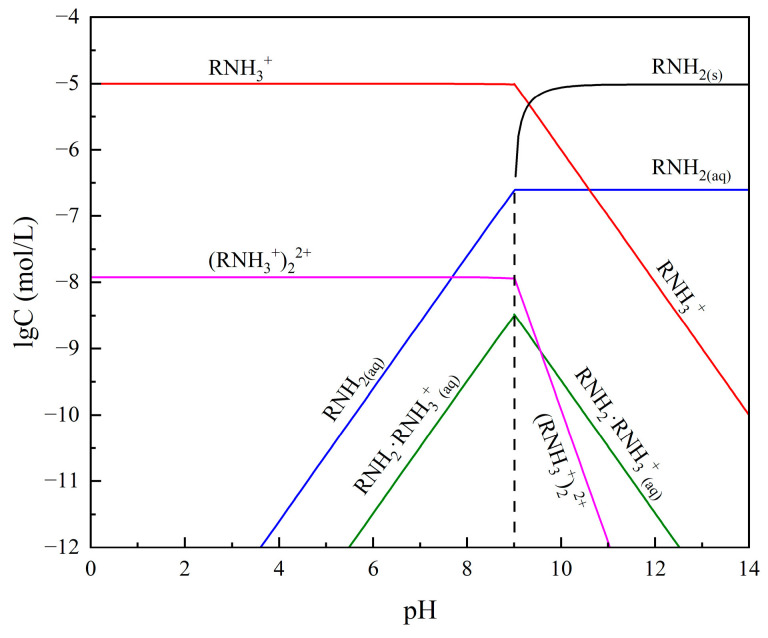
Species distribution diagram of 2 × 10^−5^ mol/L octadecyl amine solution.

Subsequently, the effect of pH on the flotation recovery of bare smithsonite and sulfidized smithsonite in the presence of 2.0 × 10^−5^ mol/L ODA was tested, and the results are displayed in Figure 5b. In the range of pulp pH from 7 to 13, the flotation recovery of smithsonite with and without sulfidization increased with the increase of pulp pH, which might be related to the present form of ODA in the different pH values (shown in Figure 6). Meanwhile, it can be seen that the flotation recovery of smithsonite was more greatly affected by the pulp pH than sulfidized smithsonite, which may be related to the fact that the surface of smithsonite is more prone to hydrolysis at lower pH and, in contrast, the generation of sulfide products slowing down the hydrolysis behavior of smithsonite surface. The above results suggested that sulfidization treatment could significantly promote the ability of ODA to collect smithsonite.

### 2.3. Effect of Sulfidization on the Adsorption Capacity of ODA

**Figure 7:**
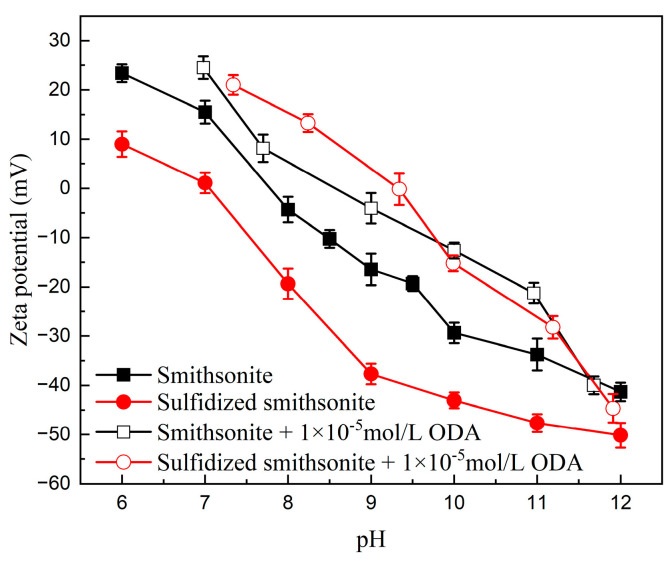
Effect of pH on the zeta potential of bare and sulfidized smithsonite before and after interaction with ODA.

**Figure 8:**
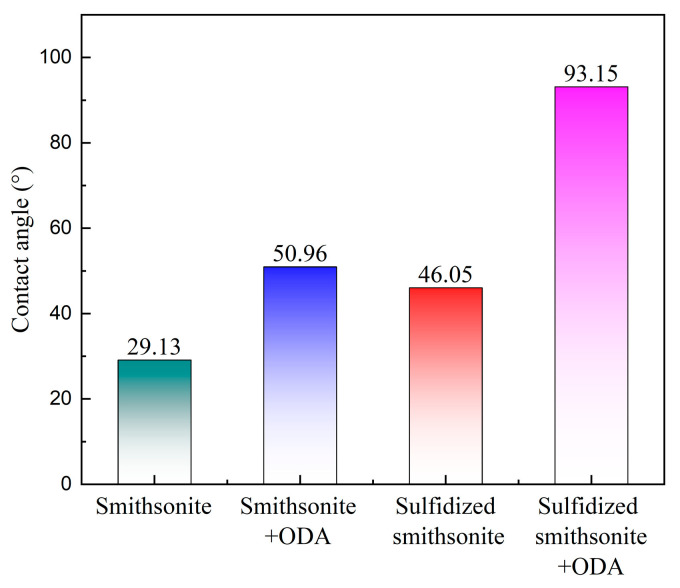
Contact angle of smithsonite under different treatment conditions.

To reveal the mechanism of sulfidization on strengthening the flotation recovery of smithsonite using ODA as a collector, the zeta potentials and contact angles of smithsonite under different reagent schemes were measured, and the results are presented in Figure 7 and Figure 8, respectively.

Figure 7 shows the changes in the surface electrical properties of smithsonite and sulfidized smithsonite with the pH value before and after the treatment of ODA. ODA could hydrolyze a variety of positively charged ammonium ions in aqueous solution, such as RNH_2(aq)_, RNH_3_^+^, RNH_2_·RNH_3_^+^_(aq)_, and (RNH_3_^+^)_2_^2+^, which will lead to an increase in the zeta potential of the mineral. It can be seen that within the measured pH range, the zeta potential of smithsonite and sulfidized smithsonite shifted positively under the action of ODA, further indicating that ODA can be adsorbed on the mineral surface before and after sulfidization. In addition, under the same pH conditions, the positive shift of the surface potential of the sulfidized smithsonite sample after the action of ODA is greater than that of smithsonite, indicating that the adsorption of ODA on the surface of sulfidized smithsonite is greater.

Figure 8 demonstrates the contact angle of smithsonite under different treatment conditions. It can be seen that the sulfidization treatment of the smithsonite surface can increase the contact angle from 29.13° to 46.05°, indicating that the generation of sulfide products on the surface of smithsonite can increase the hydrophobicity of the mineral surface to a certain extent. After the treatment of ODA, the contact angle of the smithsonite surface increased from 29.13° to 50.96°, and that of the sulfidized smithsonite surface increased from 46.05° to 93.15°, indicating that the action of ODA can improve the hydrophobicity of the mineral surface, and the sulfidization treatment is conducive to promoting the effect of ODA on the hydrophobicity of smithsonite, which was the direct reason for a preferable floatability of smithsonite.

**Figure 9:**
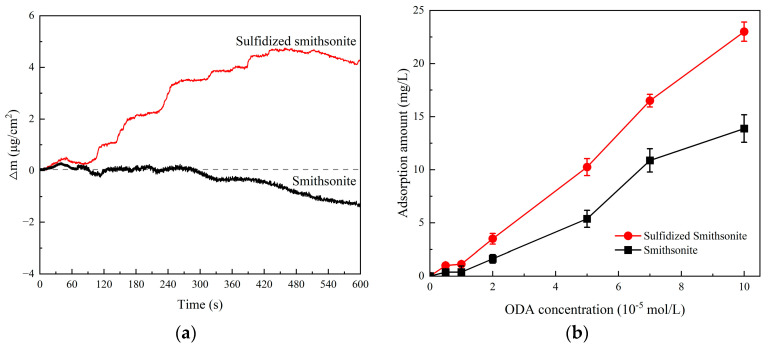
The adsorption capacity of ODA on smithsonite with and without sulfidization (**a**). QCM, (**b**). TOC.

The inference was well verified by the adsorption results that the amount of ODA shown in Figure 9 adsorbed on sulfidized smithsonite was greater than that on bare smithsonite, and it also agreed well with those of flotation and zeta potentials. Figure 9a shows the effect of ODA on the quality change in the bare and sulfidized smithsonite sample. It can be seen that the mass of bare smithsonite did not increase after the addition of ODA, and it even decreased after 300 s. This may be due to the strong solubility of smithsonite itself. At this time, even if ODA is adsorbed on the surface of smithsonite, it might be dissolved into the liquid phase along with surface dissolution. For sulfidized smithsonite, the sample mass increased significantly with time after the addition of ODA and reached the maximum value at 450 s, which might be related to the rapid adsorption of ODA on the surface of sulfidized smithsonite. Since ODA has strong adsorption capacity, the quality of the sulfidized smithsonite sample decreases after 450 s, which may be due to the attenuation of the sulfide layer. Figure 9b shows the adsorption amount of ODA on the surface of bare and sulfidized smithsonite detected by TOC. The test results show that the adsorption of ODA on the surface of bare and sulfidized smithsonite increases with the addition of ODA. Moreover, the adsorption of ODA on the surface of sulfidized smithsonite is greater than that on the surface of smithsonite, which is basically consistent with the above QCM test results, further indicating that sulfidization treatment can increase the adsorption of ODA on the surface of smithsonite, which might be beneficial to the flotation recovery of smithsonite using ODA as collectors.

### 2.4. FTIR Analysis Results

To study the mechanism of sulfidization of Na_2_S on the adsorption of ODA on the surface of smithsonite, FTIR spectra of ODA and smithsonite with and without sulfidization before and after the treatment of ODA were detected.

**Figure 10:**
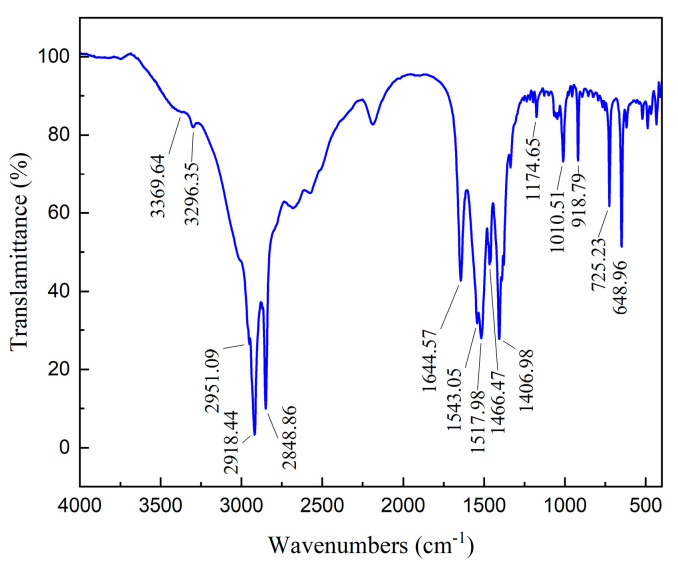
FTIR spectrum of octadecyl amine (ODA).

The spectrum of ODA is displayed in Figure 10. In the ODA spectrum, the asymmetric and symmetric stretching vibration and symmetric bending vibration absorption peaks of R-NH_2_ appeared at 3369.64 cm^−1^, 3296.35 cm^−1^, and 1644.57 cm^−1^, respectively [20]. The asymmetric (-CH_3_), asymmetric and symmetric (-CH_2_-) stretching vibration absorption peaks of C-H separately occurred at 2951.09 cm^−1^, 2918.44 cm^−1^, and 2848.86 cm^−1^. The peaks that emerged at 1517.98 cm^−1^~1543.05 cm^−1^ were assigned to the antisymmetric bending vibration absorption band of RNH_3_^+^, whereas the peaks belonging to the asymmetric bending vibration and symmetric bending vibration of -CH_3_ were recorded at 1466.47 cm^−1^ and 1406.98 cm^−1^. In addition, the peak at 1174.65 cm^−1^ was attributed to the stretching vibration of C-N. The FTIR spectra of bare and sulfidized smithsonite before and after the treatment of ODA are shown in Figure 6.

**Figure 11:**
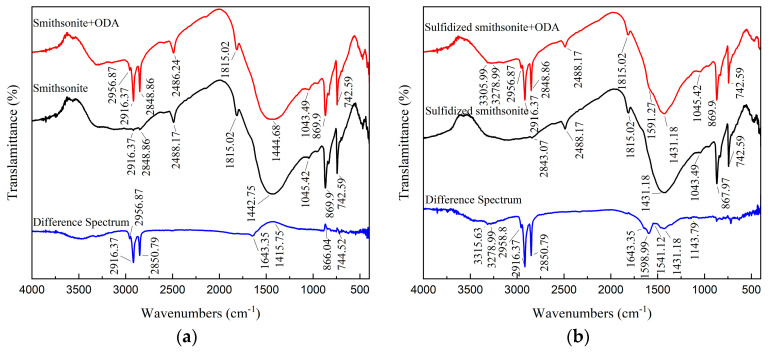
FTIR spectra of smithsonite (**a**) and sulfidized smithsonite (**b**) under different reagent schemes.

As illustrated in Figure 11, after ODA treatment, a new absorption peak at 2956.87 cm^−1^ was detected on the smithsonite surface; meanwhile, the intensity of the characteristic peaks located at 2916.37 cm^−1^ and 2848.86 cm^−1^ was significantly enhanced. The three characteristic peaks were attributed to C-H stretching vibration in ODA, and the corresponding bands of them were also no obvious shift, indicating that ODA could be adsorbed on the surface of smithsonite. In addition, using the characteristic absorption peaks at 2488.17 cm^−1^ and 1815.02 cm^−1^ in the spectrum of bare smithsonite as reference peaks, peak difference processing was performed on the FTIR of smithsonite before and after the interaction with ODA. It could be seen that a symmetrical bending vibration absorption peak on the differential spectrum appeared at 1643.35 cm^−1^, which was not apparent compared to 1644.57 cm^−1^ belonging to -NH_2_ in ODA. In addition, the relative peak intensity of the characteristic absorption peaks attributed to CO_3_^2−^ ions at 1415.75 cm^−1^, 866.04 cm^−1^, and 744.52 cm^−1^ was enhanced [19]. The above results indicated that ODA might be physically adsorbed on the smithsonite surface and thus result in more CO_3_^2−^ ions exposed on its surface.

It could also be seen from Figure 11 that for the sulfidized smithsonite treated with ODA, its FTIR spectrum was similar to that of sulfidized smithsonite, except for the three new adsorption peaks at 3305.99 cm^−1^, 3278.99 cm^−1^ (both attributed to -NH_2_) and 1591.27 cm^−1^, indicating that the interaction mechanism of ODA with sulfidized smithsonite was relatively complex. Further, the absorption peaks at 2488.17 cm^−1^ and 1815.02 cm^−1^ were used as reference peaks to perform peak difference processing on the FTIR spectra of sulfidized smithsonite before and after interacting with ODA. This indicated that the new adsorption peaks at 3315.63 cm^−1^, 3278.99 cm^−1^, 1643.35 cm^−1^, 1598.99 cm^−1^, 1541.12 cm^−1^, and 1143.79 cm^−1^ appeared on the differential spectrum, and those at 3315.63 cm^−1^ and 3278.99 cm^−1^ were shifted from the -NH_2_ (3369.64 cm^−1^ and 3296.35 cm^−1^) peaks contained in ODA. The peaks at 1598.99 cm^−1^ and 1541.12 cm^−1^ showed negative–positive deformation peaks, which might be caused by the peak shift of RNH_3_^+^ in ODA. Therefore, it could be inferred that the ODA was chemically adsorbed on sulfidized smithsonite by the action of R-NH_2_ and RNH_3_^+^.

The above FTIR results have shown that ODA adsorbed on the bare (physically) and sulfidized (chemically) smithsonite in different forms of action, which might have led to the large differences in the adsorption of ODA and flotation behavior between bare smithsonite and sulfidized smithsonite.

### 2.5. XPS Analysis Results

**Figure 12:**
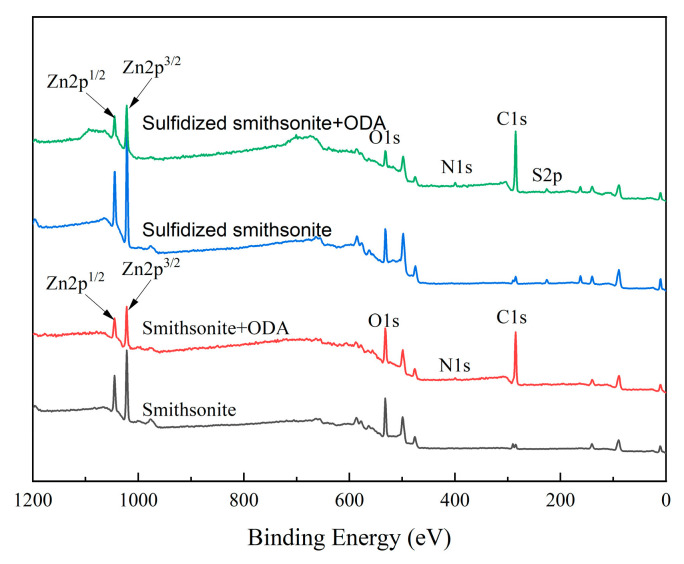
XPS survey spectra of smithsonite under different reagent schemes.

To further reveal the reaction mechanism between ODA and bare/sulfidized smithsonite, XPS experiments were performed to analyze the composition and valence change in elements on the bare/sulfidized smithsonite surface before and after ODA treatment. The XPS survey spectra and various surface elements binding energy values of smithsonite under different reagent schemes are demonstrated in Figure 12.

It could be seen from Figure 12 that compared to bare/sulfidized smithsonite, after the interaction of them with ODA, the new peak of N1s appeared in their XPS survey spectra, and the peak intensity of the C1s peak was significantly enhanced, indicating that ODA was adsorbed on both bare and sulfidized smithsonite. Furthermore, it was also found from Figure 8 that the peak intensity of N1s on sulfidized smithsonite was much stronger than that on bare smithsonite, indicating that the amount of ODA adsorbed on the sulfidized smithsonite surface, which was consistent with the results of zeta potential and adsorption.

**Figure 13:**
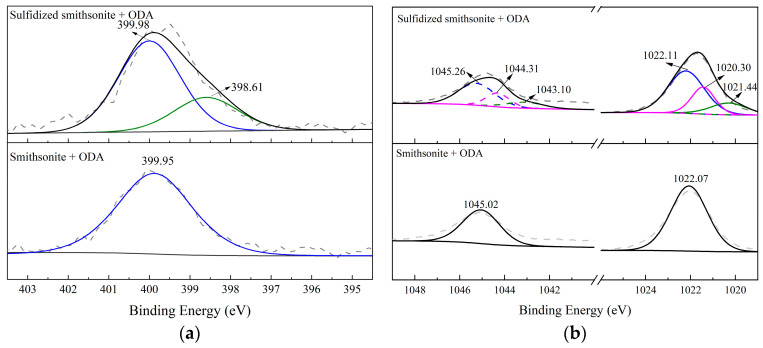
High-resolution spectra of N1s (**a**) and Zn2p (**b**) on bare/sulfidized smithsonite surface before and after the treatment with ODA.

Figure 13 shows the narrow spectra of N1s and Zn2p on bare/sulfidized smithsonite before and after the reaction with ODA. As shown in Figure 13a, it can be seen that with the treatment of ODA, a new peak attributed to the N1s of ODA with the binding energy of 399.95 eV appeared on the smithsonite surface, while the N1s peak on the sulfidized smithsonite surface could be well fitted into two peaks of 399.98 eV and 398.61 eV. The results indicated that the chemical environment of part N1s of ODA adsorbed on sulfidized smithsonite had changed obviously with the binding energy of N1s shifting from 399.98 to 398.61 eV by 1.37 eV. In addition, Figure 13b also indicated that the peak of Zn2p for smithsonite with the treatment of ODA could be fitted into double peaks at 1022.07 eV and 1045.02 eV separately belonging to Zn2p^3/2^ and Zn2p^1/2^ of smithsonite, while for Zn2p of sulfidized smithsonite treated with ODA, its binding energy shifted to a certain extent. It was found that the peak of Zn2p could be fitted into three pairs of characteristic peaks, among which the peaks at 1022.11 eV and 1045.26 eV were assigned to ZnCO_3_, 1021.44 eV and 1044.31 eV were attributed to ZnS, and new peaks at 1020.30 eV and 1043.31 eV appeared. Based on the aforementioned analysis, it is reasonable to infer that the pretreatment of sulfidization could transform the interaction mechanism of ODA on the smithsonite surface from physical to chemical by providing a beneficial environment for complexing N of ODA with Zn active sites of smithsonite [21,22].

## 3. Materials and Methods

### 3.1. Materials and Reagents

**Figure 14:**
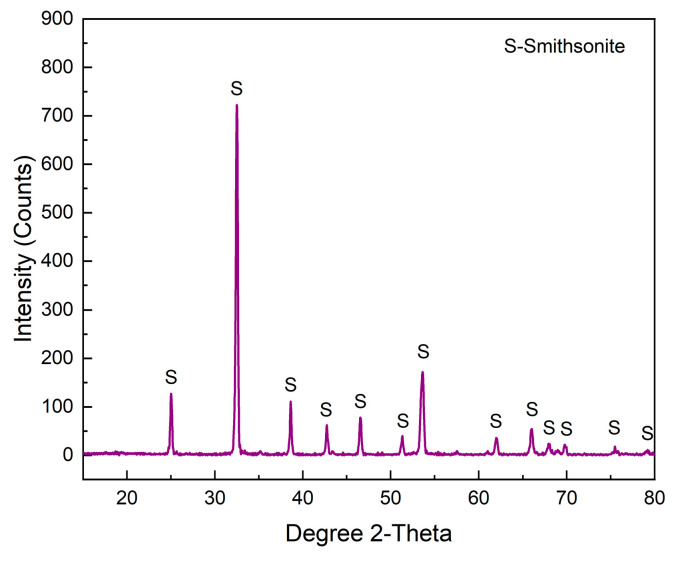
XRD pattern of smithsonite.

The single mineral of smithsonite was taken from a mine in Yunnan Province. The obtained lump ore samples were smashed with a hammer, and the mineral samples with better crystallization were hand-selected. After the samples were crushed, they were dry-milled with a porcelain ball mill and dry-sieved with a standard sieve to obtain two particle size samples of 38~74 μm and −38 μm. The 38~74 μm particle size samples were used for single mineral flotation tests, and the −38 μm particle size samples were further ground to −2 μm for qualitative analysis and testing. In addition, some −2 μm samples were further ground to −100 nm and coated on the surface of a gold-loaded quartz crystal chip for electrochemical quartz microcrystal balance testing. Figure 14 shows the XRD spectrum of the single mineral sample of smithsonite. Combined with the chemical element analysis, it shows that the purity of the smithsonite sample is as high as 96%, which meets the grade of single mineral experimental research.

The 60% purity octadecylamine acetate (ODA) used as the collectors were purchased from Beijing Bailingwei Chemical Technology Co. Ltd., Beijing, China. Sulfidized reagent using analytical-grade sodium sulfide (Na_2_S·9H_2_O) was purchased from Sinopharm Chemical Reagent Co., Ltd., Shanghai, China. Analytical-grade methyl isobutyl carbinol (MIBC), an aliphatic alcohol, used as the frother was purchased from Macklin Biochemical Technology Co., Ltd, Shanghai, China. The pH of the pulp was adjusted with hydrochloric acid (HCl) and sodium hydroxide (NaOH). Deionized water (Resistivity = 18.2 MΩ.cm) was used for all the tests.

### 3.2. Micro-Flotation Tests

The single mineral flotation test uses an improved Hallimond tube. Each time, 2 g of a smithsonite single mineral and 150 mL of deionized water containing 0 ppm of dissolved oxygen were accurately weighed and placed in a 250 mL conical flask with a glass stopper. The conical flask was sealed and stirred with a magnetic device for 1 min. The pH of the ore pulp was adjusted with hydrochloric acid and sodium hydroxide, and the sulfiding agent (Na_2_S·9H_2_O) was added and stirred for 5 minutes. Thereafter, the collector (ODA) and frother (MIBC) were added in sequence and stirred for 3 minutes and 1 minute, respectively. After the ore pulp had settled for 30 seconds, the precipitate was transferred to a Hallimond tube, and a small amount of supernatant was added to maintain the liquid level in the tube. At the same time, the magnetic stirrer (speed of 500rpm) was turned on, and flotation was carried out for 3 min with a constant nitrogen gas flow of 150 mL/min. The obtained foam product and the product in the tank were used as the flotation concentrate and tailings, respectively. The flotation recovery rate was calculated after drying and weighing.

### 3.3. Contact Angle Tests

The contact angle test is mainly used to characterize the macroscopic differences in the wettability of mineral surfaces under different experimental conditions. First, small pieces of smithsonite with suitable particle size and good crystallinity were selected and embedded in epoxy resin; then, diamond grinding wheels with roughness of 100 µm, 40 µm, and 9 µm and alumina powders of 1.0 µm, 0.3 µm, and 0.05 µm were used to grind and polish to obtain a flat and smooth surface and then rinsed with deionized water several times and dried for use. The samples were treated according to the same reagent conditions and action time as the flotation conditions. Then, the contact angle of the mineral surface was measured by the drop method using a fully automatic contact angle meter. (JC2000D, Zhongchen Digital Tech. lim. Shanghai, China).

### 3.4. Zeta Potential Measurements

The zeta potential of the mineral surface was measured by a Coulter Delsa 440sx Zeta analyzer instrument (Beckman Coulter, Brea, CA, USA), and 0.01 mol/L potassium chloride solutions with different dissolved oxygen contents were prepared before measurement. In each test, 20 mg of the sample to be tested was stirred with 40 mL of potassium chloride solution with a specific dissolved oxygen content for 1 min to stabilize the system; then, after adjusting the pH of the slurry, the corresponding reagents were added in sequence and stirred for a time corresponding to the flotation slurry adjustment process. After that, the suspension was settled for 3 min, and the supernatant was injected into the electrolytic sample cell for zeta potential determination. Each sample was measured 3 times under the same test conditions, and the average value was taken.

### 3.5. ODA Adsorption Tests

An electrochemical quartz crystal microbalance (eQCM 10M, Gamry Instruments, Warminster, PA, USA) was used to monitor the dynamic response of mineral particle mass and potential under various conditions. The smithsonite quartz crystal core (Sm-Au-QC) was prepared by a sputtering process. Nano-scale smithsonite, binder Nafion perfluorosulfonic acid (PFSA) polymer and anhydrous ethanol were mixed and stirred in a certain proportion for 2 h, sputtered onto the surface of Au-QC with a center frequency of 5 MHz, and then low-temperature dried. The octadecane adsorption test was carried out with the help of a total organic carbon analyzer (Shimadzu TOC-V-CPH, Tokyo, Japan), and the residual concentration method was used to determine the adsorption of ODA on the mineral surface under different slurry conditions. Each time, 1 g of the single mineral sample to be tested was weighed and added to a 50 mL conical flask, and 40 mL of deionized water with a specific dissolved oxygen content was added. After the reaction under the required conditions, it was allowed to stand for 1 min, the upper suspension was extracted and centrifuged at 9000 r/min for 10 min, and the supernatant was extracted after centrifugation for total organic carbon determination. When the concentration was too high, the supernatant must be further diluted before determination. The concentration difference before and after the determination is the amount of adsorption of the agent on the mineral surface.

### 3.6. FTIR Analysis

An FTIR infrared test mainly characterizes the microscopic changes of flotation reagent adsorption on the surface of mineral particles from a microscopic perspective. Each time, 1 g of pure mineral sample (−2 μm) was weighed and placed in a 50 mL conical flask, and an appropriate amount of distilled water and reagent was added and stirred for 30 min. A centrifuge (6000 rpm) was used for solid–liquid separation, and the obtained ore sample was washed 3 times with distilled water of the same pH value. Finally, the prepared sample was placed in a vacuum oven and dried at 40 °C. For infrared spectrum detection, we used a potassium bromide tablet method. Each time, 2 mg of ore sample was taken and mixed with 200 mg of potassium bromide, and then the sample was pressed into tablets after grinding to a certain fineness. Then, the sample was placed in a Fourier transform infrared spectrometer for detection. Then, the sample was performed using a Spectrum One FTIR spectrometer (Version BM, PerkinElmer, Waltham, MA, USA). 

### 3.7. XPS Analysis

X-ray photoelectron spectroscopy (XPS) is mainly used to characterize the changes in the valence state of elements on the surface of minerals and to measure the difference between the adsorption of ODA and the sulfidization reaction on the surface of minerals. The test was carried out on the ESCALAB 250Xi X-ray photoelectron spectrometer (Thermo Fisher Scientific, Waltham, MA, USA). First, we weighed 1 g of pure mineral sample smithsonite into a 50 mL conical flask; then, we added ultrapure water and stirred for 2 min. Then, after adding flotation reagents and fully reacting, a centrifuge was used for solid–liquid separation, and the obtained ore sample was washed 3 times with distilled water of the same pH value. Finally, the prepared sample was placed in a vacuum oven and dried at 40 °C. All binding energies were calibrated to 284.8 eV using C1s.

## 4. Conclusions

In the current research, the effect of sulfidization on the adsorption of ODA and its response to floatability were tested by zeta potential, contact angle measurement, total organic carbon analyzer (TOC), quartz micro-crystalline balance (QCM), Fourier transform infrared spectroscopy (FTIR), X-ray photoelectron spectroscopy (XPS), and micro-flotation tests. Based on the experimental results, the following conclusions could be drawn:

(1) The pretreatment of sulfidization could generate the sulfide product in the form of ZnS on the smithsonite surface, which was unevenly distributed and prone to form on the fracture zone and fine particles.
(2) Micro-flotation tests suggested that the treatment of sulfidization could improve the floatability of smithsonite, and the treatment of sulfidization is able to increase the flotation recovery from 41.6% to 64.3% at the ODA concentration of 2 × 10^−5^ mol/L.
(3) Adsorption tests exhibited a preferable adsorption capacity toward sulfidized smithsonite compared to bare smithsonite, thus rendering better hydrophobicity and floatability.
(4) FTIR and XPS further indicated that the interaction mechanisms of ODA on bare (physical) and sulfidized smithsonite (chemical) surfaces were different. The pretreatment of sulfidization provided a beneficial environment for complexing N of ODA with Zn active sites on the smithsonite surface, leading ODA more firmly to adsorb on smithsonite.

## Figures and Tables

**Table 1:** Changes in the atomic abundance and binding energy before and after sulfidization.

	Samples	Atomic Orbitals			
		Zn2p	C1s	O1s	S2p
Relative abundance (%)	Smithsonite	20.96	28.90	50.14	/
	Sulfidized smithsonite	29.89	23.73	32.09	14.29
	Difference	8.93	−5.17	−18.05	/
Binding Energy (eV)	Smithsonite	1022.04	289.91	532.04	/
	Sulfidized smithsonite	1021.59	290.06	531.79	161.86
	Chemical shift	−0.63	0.15	−0.25	/

## Data Availability

The original contributions presented in the study are included in the article, further inquiries can be directed to the corresponding author.
